# High Accuracy Open-Type Current Sensor with a Differential Planar Hall Resistive Sensor

**DOI:** 10.3390/s18072231

**Published:** 2018-07-12

**Authors:** Sungho Lee, Sungmin Hong, Wonki Park, Wonhyo Kim, Jaehoon Lee, Kwangho Shin, Cheol-Gi Kim, Daesung Lee

**Affiliations:** 1 Korea Electronics Technology Institute, Gyeonggi 13488, Korea; smhong@keti.re.kr (S.H.); wkpark74@keti.re.kr (W.P.); kimwh@keti.re.kr (W.K.); leeds@keti.re.kr (D.L.); 2 Department of Emerging Materials Science, DGIST, Daegu 42988, Korea; ljh@dgist.ac.kr (J.L.); cgkim@dgist.ac.kr (C.-G.K.); 3 Department of Information and Communication Engineering, Kyungsung University, Busan 608-736, Korea; khshin@ks.ac.kr

**Keywords:** current sensor, high accuracy, magnetic sensor, planar hall resistive (PHR), post-CMOS process, read-out IC (ROIC)

## Abstract

In this paper, we propose a high accuracy open-type current sensor with a differential Planar Hall Resistive (PHR) sensor. Conventional open-type current sensors with magnetic sensors are usually vulnerable to interference from an external magnetic field. To reduce the effect of an unintended magnetic field, the proposed design uses a differential structure with PHR. The differential structure provides robust performance to unwanted magnetic flux and increased magnetic sensitivity. In addition, instead of conventional Hall sensors with a magnetic concentrator, a newly developed PHR with high sensitivity is employed to sense horizontal magnetic fields. The PHR sensor and read-out integrated circuit (IC) are integrated through a post-Complementary metal-oxide-semiconductor (CMOS) process using multi-chip packaging. The current sensor is designed to measure a 1 A current level. The measured performance of the designed current sensor has a 16 kHz bandwidth and a current nonlinearity of under ±0.5%.

## 1. Introduction

Recently, the need for high accuracy current sensors has increased for the automobile industry and smart grid applications [1,2,3,4,5,6,7,8]. In the automobile industry, traditional engine-based cars as well as electric cars need accurate current sensors to control various motors, such as electric motors, electric power steering motors, and brake motors. Furthermore, the smart grid industry needs a variety of high accuracy current sensors to measure the quality and quantity of the power consumption in the facilities of factories. Many IoT (Internet of things) devices are accelerating the necessity of smart small-power metering. Current can be obtained by measuring the voltage drop using a high accuracy shunt resistor. However, the applicable voltage range is limited and the voltage drop is rapidly increased along with the increasing current. As a non-contacting method, many conventional current sensors have utilized current transducers with ferromagnetic-based material by wrapping around the current carrying conductor and leaving a small air gap (typically 2–5 mm) [3,4,5,6,7,8,9,10,11,12]. Due to the properties of ferrimagnetic material, such a structure shows a strong magnetic gain and robustness against crosstalk. However, the size and weight have to be increased according to the magnitude of the measuring current, which makes it hard to install and maintain metering systems in which the installation place of the sensor is normally limited. As an alternative solution, current transformers using an optical signal can be used by utilizing the variation of the optical signal to the magnetic force [13,14,15]. Although the optical current sensing method is a candidate for good reliability and sensitivity, the birefringence in the optical cable is prone to vary according to temperature variations.

**Figure 1:**
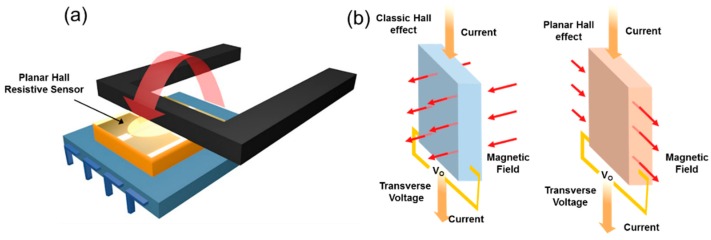
(**a**) Structure of an open-type current sensor and (**b**) mechanism of Planar Hall Resistive (PHR) sensor.

On the other hand, open-type current sensors based on the magnetic sensor directly utilize the magnetic field induced from a current-flowing conductor, as shown in Figure 1a. The open-type current sensor has many advantages because its structure provides a small size, low cost, a small form-factor and a configurable sensing current level according to the distance to a current-flowing conductor. However, the limited sensitivity of the magnetic sensor may degrade the accuracy of the current sensor. Although the sensor sensitivity can be increased by adding an integrated magnetic concentrator (IMC) using ferromagnetic material, the additional layer has the disadvantage of increasing the overall cost. Moreover, the IMC is sensitive to temperature variations. Another challenge of the open-type structure is that the open-type structure of the sensor is so vulnerable to an external magnetic field that the sensing magnetic field is highly affected by magnetic interference due to adjacent conducting lines [16,17]. Recently Allego demonstrated a 2.5 A current sensor using a differential structure with two Hall sensors in a commercial product [18]. However, the differential structure does not utilize the best sensing position considering the magnetic field distribution, because normally the maximum point of the magnetic field does not become the next of the conductor line, but the center of the conductor. Moreover, when the required measuring current level is low, for example, a 1 A level, the high accuracy specification of the current sensor becomes very high and extremely hard to achieve [19,20,21,22].

In this paper, we propose a high accuracy open-type current sensor for a 1 A current level using Planar Hall Resistive (PHR) sensor. The origin of the PHR is from anisotropic scattering of electrons carrying current due to the magnetic moment of the lattice atoms. On the other hand, the ordinary Hall effect is caused by the Lorentz force interaction between charged particles moving in a magnetic field, as shown in Figure 1b [23]. Without using an IMC layer, we utilize two highly sensitive PHR sensors to increase the sensitivity, forming a differential magnetic sensor. The PHR sensor achieves high sensitivity with stable temperature characteristics. In addition, we develop a new Read-Out integrated circuit (ROIC) for the PHR sensors. The differential sensor and ROIC are attached together by a post-Complementary metal-oxide-semiconductor (CMOS) process. The dedicated current path for a 1 A current level is patterned on a printed circuit board beneath the bottom of the ROIC in a package. In the following sections, we describe the structure, design and performance of the proposed current sensor.

## 2. Design and Structure of Current Sensor

### 2.1. Differential Structure

**Figure 2:**
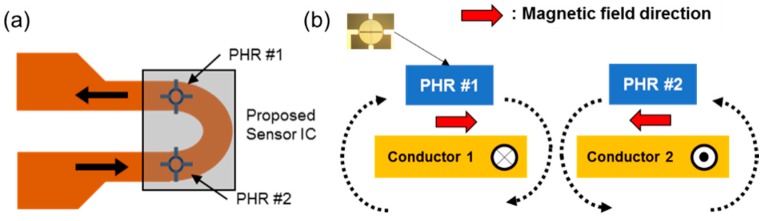
(**a**) The proposed differential structure with two PHR sensors and the directions of the magnetic field and (**b**) the location of the current sensors on a current-flowing conductor.

Figure 2 shows the proposed differential sensor structure. Two neighboring PHR sensors are placed on the corresponding conductors in the opposite current direction, as shown in Figure 2a. Thus, when current starts to flow through a conducting line, each PHR sensor receives a magnetic flux in the opposite direction, as shown in Figure 2b.

The output voltage difference between two PHR sensors linearly corresponds to the sensed current value. The proposed differential configuration has three main advantages. First, when an external magnetic field interferes with the current sensor, the unwanted magnetic field influence the same effect to two PHR sensors. Thus, the magnetic interference to the two PHR sensors can be cancelled out by exploiting the differential output signals. As a result, the effect of the interfering force of the magnetic field comes to a negligible level. Second, when the differential sensor data is utilized from the two sensors, the sensitivity of the current sensor is improved, because the sensitivity of the differential structure is twice that of one PHR sensor. Even though two PHR sensors are unmatched, the overall sensitivity can be increased. However, the cancellation degree of the common mode perturbation can be reduced depending on the unmatched degree. The mismatch between two sensors can be calibrated by a piecewise linear method or an auto-tuning circuit [24]. Generally, an extra process cost or an IMC layer is required to increase the sensitivity of the magnetic sensor. Third, the sensor can be located close to the center of the conductor line where maximum magnetic field is appeared because the PHR sensor can sense horizontal magnetic field. Therefore, the sensor can receive more of a magnetic field, which is different form the case in the commercial sensor [18].

**Figure 3:**
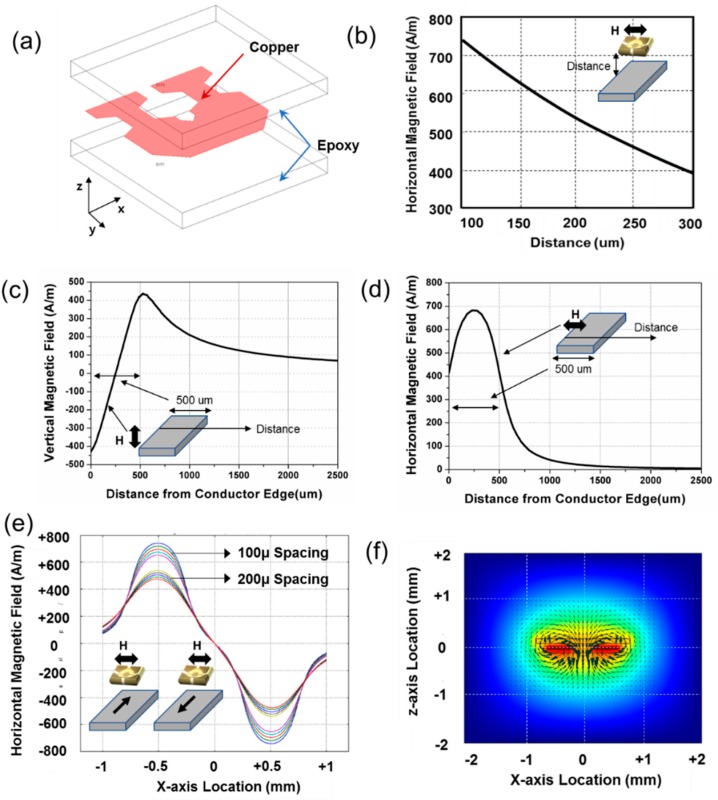
(**a**) The pattern of the current-flowing conductor line, (**b**) a simulated magnetic field distribution image around the conductor lines. (**c**) Vertical magnetic field and (**d**) horizontal magnetic field according to the distance between the PHR sensor and the conductor line, and (**e**) horizontal magnetic field according to the distance between the conductor line and a sensor. (**f**) Horizontal magnetic field of two conductor lines in opposite current direction.

The relation between the constant current (*I*) through a conductor line and the magnetic flux (*B*) is as follows:

(1) $$B=\frac{\mu_{0}\mu_{r}I}{4\pi }\int \frac{dl\times \hat{r}}{r^{2}}=\frac{\mu_{0}\mu_{r}I}{4\pi }\cdot \frac{1}{r},$$

where $\mu_{0}$ and $\mu_{r}$ are the vacuum permeability and relative permeability, respectively. And r denotes the spacing between the conductor and the sensing location. The spacing of the differential structure is one of the most important specifications. Optimized distances between the PHR sensor and the conductor line and between two conductors are essential to achieve high performance. The distances are determined through intensive magnetic simulations, as shown in Figure 3.

First, the distance between a PHR sensor and the corresponding conductor line determines the maximum magnitude of the magnetic flux. As the distance becomes closer, the magnetic field-current conversion coefficient increases. Normally, the thicknesses of the ROIC and the conductor line determine the minimum distance. In this design, a copper line with a thickness of 35 μm and a ROIC with a thickness of 80 μm are employed, as shown in Figure 3a. The resultant distance becomes 150 μm, including epoxy layers for isolation. These dimensions are selected to flow 1 A current safely through a conductor considering the overall size and self-heating conditions. Figure 3b shows the maximum magnetic field becomes 600 A/m and the corresponding magnetic field-current conversion coefficient is 7.5 G/A at this distance. Although the distance can be reduced by placing the conductor line on the ROIC, patterned conductor lines on a printed circuit board are employed to obtain a low process cost.

Second, the spacing between two conductor lines is dependent on the entire package size and the degree of interference of the magnetic force from the other side. Figure 3c,d shows the vertical and horizontal magnetic field distributions along with the x-axis, respectively when 1 A current is flowing through only one conductor line with a 500 μm width. When the location is more than the width of the conductor (=500 μm), the magnetic field drops rapidly by Ampere’s law. The zero in the x-axis in Figure 3c,d means the side of the outermost conductor. Figure 3c indicates that the magnitude of the vertical magnetic fields at both sides (0 and 500 μm) of the conductor is the same with the opposite polarity. The peak value of the horizontal magnetic field is more than that of the vertical magnetic field. Under two conductor lines in opposite current direction, the sensitivity from the differential structure can be reduced because the magnetic field from the neighboring conductor line interfere the detectable magnetic force. We designed the spacing of 500 μm between two conductors because the magnetic field decreases to under 10% compared to the maximum, as the distance becomes more than 1000 μm. Figure 3e,f shows the magnetic field curve in x-axis and the graphical distribution image of the designed dimension.

**Figure 4:**
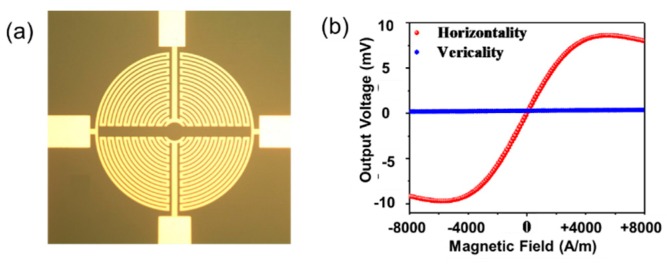
(**a**) A photograph of the PHR sensor and (**b**) the relation between the output voltage and the applied magnetic field with horizontality or verticality.

Figure 4 shows the microphotograph of the PHR sensor and its performance. This PHR sensor has several layers consisting of Fe∙Mn, Cu, Ni∙Fe and passivation layers so that the sensitivity can be maximized [25]. The diameter and the number of turns of the PHR sensor affect the sensitivity and the dynamic range of the current sensor. As the number of turns is increased, the sensitivity is increased and the dynamic range is reduced. Therefore, the number of turns is increased as long as the dynamic range is permitted with a design margin. The large diameter also increases the sensitivity of PHR sensor. However, the diameter also decides the overall sensor area, so that the diameter is designed considering the overall width of the ROIC. The diameter of the sensor was designed to be 440 μm and the number of turns was set to 19, as shown in Figure 4a. The designed current conductor generates not only the horizontal magnetic field, but also vertical magnetic field. However, the adopted PHR sensor does not respond to the vertical magnetic field. Figure 4b shows that the sensor only senses the horizontal magnetic field and does not respond to the perpendicular field. The PHR sensor can be modelled as a bridge-type resistor network. In this design, the branch resistance of the bridge network is designed to be about 1 Kohm, which is optimized for current consumption and sensitivity.

### 2.2. Design of Current Sensor

**Figure 5:**
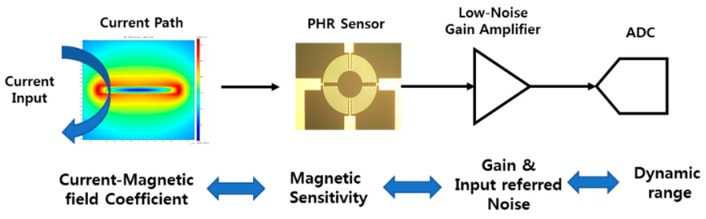
Design parameters in the signal chain of the current sensor.

In the design of the current sensor including a conductor line, the current-to-magnetic field conversion coefficient, the magnetic sensitivity, the gain and the noise of the ROIC need to be carefully chosen with the resolution and the dynamic range of the ADC in the ROIC, as shown in Figure 5 [26].

Table 1 shows the main design parameters of the proposed current sensor. In the last stage, the overall gain of the signal chain needs to be designed to accommodate the maximum range and minimum detectable signal of the analog-to-digital converter (ADC). For a 0.1% accuracy of the 1 A current sensor, a 1 mA current level should be detectable. For a 1 mA current, the output of the differential PHR sensor becomes 12 μV in this design, which is larger than the input referred noise of the ROIC (=2.4 μV). For the maximum acceptable range, the input signal of the ROIC becomes 360 mV when the measured current becomes 1 A current, which is satisfied by the dynamic range of the ADC in the ROIC.

**Figure 6:**
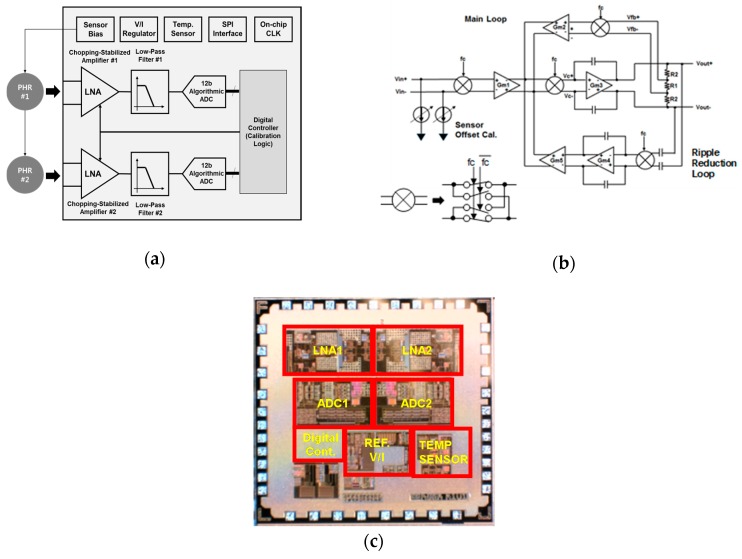
(**a**) A block diagram of the ROIC, (**b**) a schematic of the front chopping-stabilized amplifier of the ROIC, and (**c**) the fabricated chip microphotograph.

The ROIC needs to amplify the incoming signal linearly from the two PHR sensors, because the output signal of the sensor is very small (~few mV). The overall structure of the ROIC is shown in Figure 6a. To process the two differential signals, two identical signal processing blocks consisting of a low noise amplifier (LNA), a low-pass filter and a 12-b ADCs are employed. The maximum voltage gain of the LNA is set be 30 dB. Figure 6b shows the schematic of the LNA which, consists of a two-stage amplifier. The feedback circuit with Gm2 consists an indirect current feedback instrumentation amplifier. The high open-loop gain of the amplifier ensures that the output current of Gm1 cancels that of Gm2, so that Vin is equal to Vfb. Thus, Vout will be equal to Vin × Gm1/Gm2 × (R1 + 2 × R2)/R1 [27]. The LNA utilizes a chopping-stabilized structure to shift flicker noise to the high frequency region. To suppress the unwanted large voltage ripple from the offset at the output of the LNA, an extra ripple reduction loop with Gm4 and Gm5 is employed [27]. With a 100 kHz chopping frequency, the flicker noise is shifted to a high frequency and filtered-out by the following low-pass filter. The width and the length for the chopping switch are sized to 1 µm and 0.18 µm to minimize the channel charge injection. The flicker noise as well as thermal noise of the amplifier need to be minimized so 3 mm large-width NMOS transistors are used at the input differential pair. The simulated input referred noise was 24 nV/sqrt(Hz). The low-pass filter utilized the second-order active Sallen-Key topology. The ADC is another important block to determine the overall performance. A 12-b algorithmic structure without a power-hungry sample-and-hold amplifier is adopted to minimize the power consumption [28]. The frequency of the ADC input clock is 4 MHz, which is generated by an on-chip clock generator. To suppress the offset voltage of the PHR sensor, digitally controlled current paths are added for calibration at the input stage. Additionally, to compensate for the temperature variation of the sensor, the bias voltage of the PHR sensor is controlled to compensate for the temperature variation by an internal regulator. The digital controller collects the data of two ADCs and the outputs for the final current data, calibrating the operation of offset and temperature. Figure 6c shows a microphotograph of the ROIC. The IC is fabricated in a standard 180 nm mixed-mode CMOS process. The ROIC chip size is 2 × 2 mm^2^. There is no external circuit except for the PHR sensors. A serial-to-parallel interface is used for the communication to the external block.

## 3. Implementation and Measurement Results

**Figure 7:**
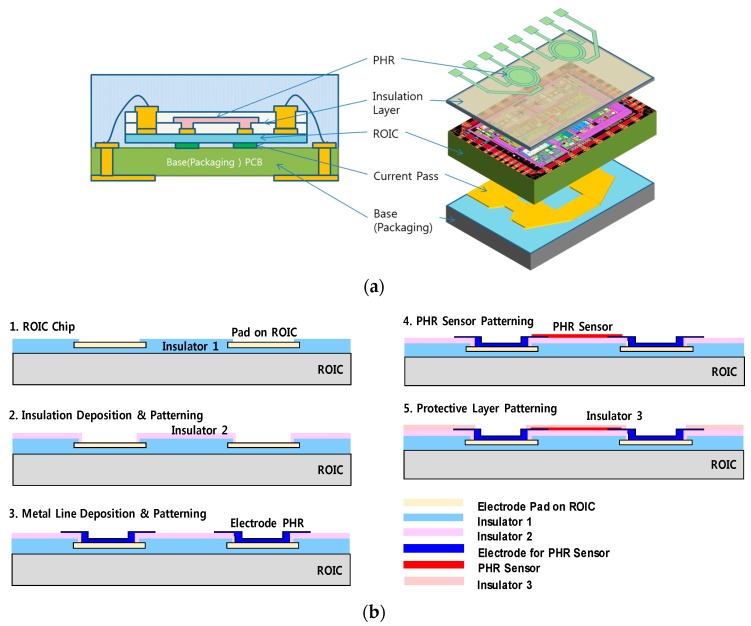

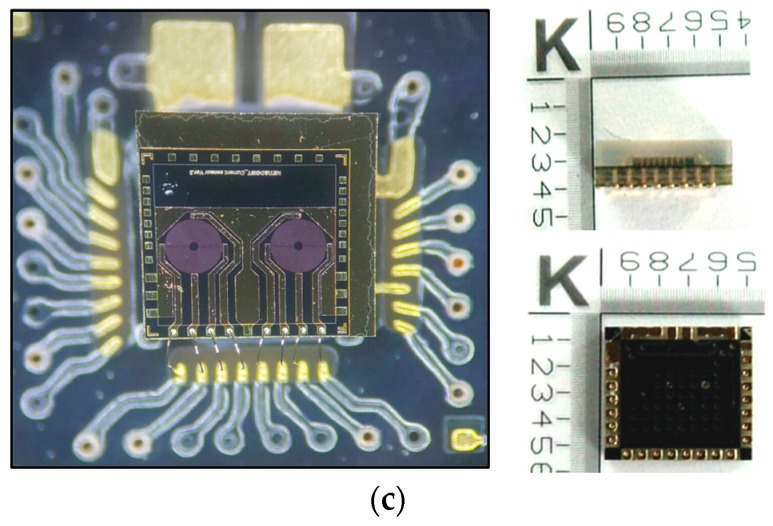
(**a**) The multi-chip structure of the current sensor, (**b**) the post-CMOS process procedure, and (**c**) a photograph of the fabricated current sensor.

A post-CMOS process was established to connect the differential PHR sensor and the ROIC, which are placed on the upper side. Figure 7a shows the several packaged stacks for the proposed current sensor. The conductor path in copper was placed symmetrically below the ROIC. The PHR sensor was patterned on the ROIC as a thin film form. Although the conductor path can be implemented on the PHR sensor, we designed a pattern on a substrate in a package for simple fabrication and cost. To combine the PHR sensor and the ROIC in the CMOS process, a few steps were necessary, as shown in Figure 7b. First, an extra insulation layer with SiO_2_ was deposited and patterned on the ROIC except for the pads because the surface of the original ROIC was not smooth enough. Then, deposition and patterning of Cr/Au (200 Å/2000 Å) for metal lines were done around the pads of the ROIC so it could be connected to the PHR sensor. To form the PHR sensor, a lift-off process was employed because the sensor was made of chemical materials instead of an etching process of metal with thin-film. Therefore, deposition was first conducted through a sputtering process after a negative photo-resist was used on the patterned ROIC. After the deposition, the negative photo-resist and unused PHR materials were removed using an acetone solution. After the PHR sensor was overlapped on the metallized pattern, another insulation layer (SiO_2_) was added for protection. The post-CMOS process was conducted at 5 mm × 5 mm chip level. The overall size of the current sensor was 4 mm × 4 mm, including routed metal patterns on the printed board in a quad flat no-lead (QFN) package. The Figure 7c shows a photo of the current sensor after these processes. To measure the performance of the current sensor, the effect of the external magnetic field was measured.

**Figure 8:**
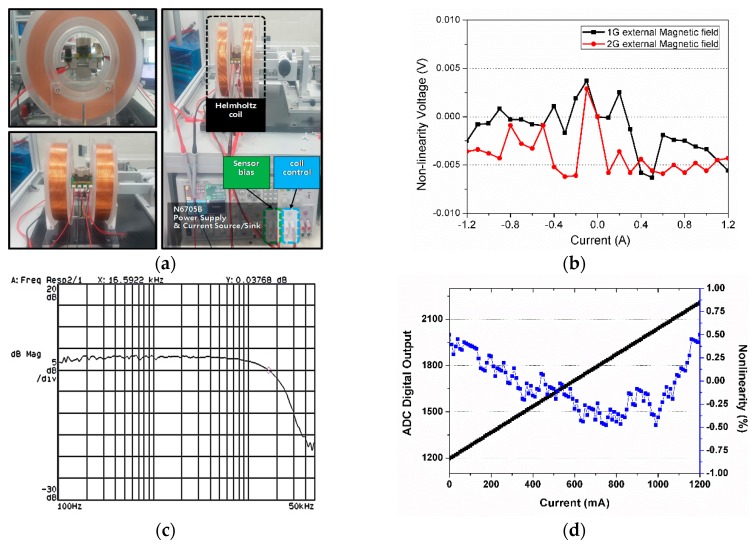
(**a**) The test setup with a Helmholtz coil, (**b**) the measured nonlinearity under an external magnetic field, (**c**) the measured frequency response, and (**d**) the measured current linearity.

Figure 8 shows the measurement setup with a Helmholtz coil. By controlling the current through the coil, the magnitude of the magnetic field could be varied. The external magnetic field with 1 Gauss and 2 Gauss was projected to the current sensor to verify the effect of the differential structure. The deviation voltage at the input of the ADC in the ROIC from no external magnetic field is shown in Figure 8b. The deviated voltage due to the external magnetic field was almost cancelled by the differential structure. The maximum resulting deviated voltage was 6 mV, which is equivalent to 9 mA input current. The offset voltage from the mismatch of two PHR sensors during the fabrication process is cancelled by two ways. First, digitally controlled current path in the ROIC is connected to the PHR sensor to cancel the offset voltage at a coarse level. Second, the remaining offset is measured and stored in a memory in a digital block. Then, the stored offset can be employed to calculate the final current data.

The frequency response of the current sensor was measured. An extra current generator board was used to generate a high frequency current waveform. The Helmholtz coil was utilized to control the magnetic field according to the frequency variation, and the output of the sensor was connected to a dynamic signal analyser. While the input signal was swept from 100 Hz to 50 kHz, the frequency response of the output was plotted, as shown in Figure 8c. The −3 dB frequency was about 16 kHz due to the limited bandwidth of the front LNA of the ROIC, not the PHR sensor. The bandwidth can be extended by increasing the current of the ROIC. The current sensing range was measured by collecting the digital data of the ADC in the ROIC according to the magnitude of the current. The nonlinearity of the current sensor was under ±0.5% and the maximum current range was 1.2 A, as shown in Figure 8d. The 0.5% accuracy can be corresponding to 1200 mA × 0.5/100 = 6 mA, when the maximum capacity of the current sensor is set to 1200 mA. The nonlinearity limitation seems to come from the nonlinear transfer function of the PHR sensor, which can be improved by a linear-fitting calibration. Table 2 shows the performance comparison with state-of-art commercial products. The proposed current sensor is the first sensor using the PHR sensor and shows comparable performance without calibration.

## 4. Conclusions

In this paper, we presented a high accuracy current sensor with a differential Planar Hall Resistance (PHR) sensor. We proposed a differential structure with two PHR sensors to reduce the interference of the magnetic field through extensive magnetic field analysis and optimization. Further, we employed a newly developed planar Hall resistance sensor instead of using a conventional Hall sensor with a magnetic concentrator. The PHR sensor and read-out IC were integrated using multi-chip packaging through a post-CMOS process. We believe that this is the first work that shows a single-chip current sensor using the PHR sensor. The current sensor was designed to measure a 1 A current level. The measured results demonstrated a 16 kHz signal bandwidth and a ±0.5% level accuracy.

## Figures and Tables

**Table 1:** Main design parameters in the current sensor.

Design Parameter	Specification
Current-to-magnetic field coefficient	7.5 G/A
Sensitivity of differential PHR sensor	1.6 mV/G
Input referred noise of read-out IC (ROIC)	24 nV/$\sqrt{\mathrm{Hz}}$
Input referred noise at ROIC for signal bandwidth	2.4 μV (Bandwidth = 10 kHz)
Voltage gain of front-end amplifier	30 dB
Achievable maximum sensed signal at analog-to-digital converter (ADC)	360 mV
ADC dynamic range (Signal-to-Noise Ratio)	70 dB
Current Consumption	4 mA @3.3 V

**Table 2:** Performance comparison to commercial products.

	DHAB S/160 [29]	TLI4970 [30]	ACS70331 [18]	This Work
Structure	Current transducer	Open-type (Differential)	Open-type (Differential)	Open-type (Differential)
Magnetic sensor	Hall	Hall	GMR	PHR
Measuring current	30 A	25 A	2.5 A	±1.2 A
Sensitivity	40 mV/A	12.5 mA/LSB (13 bit)	800 mV/A	1.2 mA/LSB (12 bit)
Nonlinearity	3.3%	1%	2.0%	0.5%
Bias voltage	5 V	3.3 V	3.3 V	3.3 V
Switching frequency	-	-	-	100 kHz
Current consumption	15 mA	mA	mA	4 mA
Power consumption	75 mW	40 mW	14.9 mW	13 mW
Resolution	62.5 mA	12. 5 mA	5 mA	5 mA
Output type	Analog	Digital	Analog	Digital
Size (Chip Size)	Large (52 mm × 34 mm)	Small (7 mm × 7 mm)	Small (3 mm × 3 mm)	Small (5 mm × 5 mm)
